# Cell therapy in renal ischemia/reperfusion experimental model using recombinant G-CSF

**DOI:** 10.1186/1753-6561-8-S4-P25

**Published:** 2014-10-01

**Authors:** Vinicius Correa Rodrigues, Isabela Bastos Binotti Araújo, Rosiane Ervati, Sônia da Penha Silva Oliveira, Breno Valentim Nogueira

**Affiliations:** 1 PG Biotecnologia, UFES, Vitoria, Brasil; 2 Departamento de Morfologia, UFES, Vitoria, Brasil

## Introduction

The colony stimulating factor granulocyte (G-CSF) is a glycoprotein capable of promoting the survival, proliferation and differentiation of hematopoietic cells. Studies demonstrate the cytoprotective action of G-CSF against renal injury by ischemia/reperfusion injury in murine models. But the literature is still controversial in relation to the risk of worsening renal function after useG-CSF, which motivated the study to elucidate possible interference on the renal function of the treated animals.

## Objective

Evaluate the renal function of rats after treatment with G-CSF at different doses of the drug.

## Methods

Male Wistar rats (n = 18), 200g approx. (CEUA050/2013), divided into 3 groups (6 animals each) : control group (C) 5% glucose solution (solvent) groups treated with G-CSF at a dose of 10 (G10) and 50 (G50) mg / kg / per 5 days. After treatment, the rats were placed in metabolic cages for urine collection and obtaining urine volume. Values were obtained from creatinine, proteinuria, urea and number of circulating leukocytes. The results were expressed as mean ± SEM. The averages ofvalues between groups were calculated using one - way ANOVA followed by post hoc Fisher for comparison between differentgroups.

## Results

A significant increase in the number of circulating leukocytes in animals treated with G- CSF (C = 9687 ± 899 / mm3;= 14375 ± 1967/mm3 G10, and G50 = 19670 ± 1663/mm3, p < 0.05). There was not a significant increase in urine volume after 24 hours treatment with G-CSF. There was no significant difference between the values of creatinine clearance, proteinuria and Urea, among groups C, G10 and G50.

## Conclusion

There was no impairment of renal function in animals treated at doses of 10 and 50 mg / kg / per 5 days.

Financial support: FAPES
